# Recent Update on Retinoic Acid-Driven Initiation of Spermatogonial Differentiation

**DOI:** 10.3389/fcell.2022.833759

**Published:** 2022-03-16

**Authors:** Indrashis Bhattacharya, Partigya Sharma, Shriya Purohit, Sachin Kothiyal, Moitreyi Das, Arnab Banerjee

**Affiliations:** ^1^ Department of Zoology, HNB Garhwal University, A Central University, Srinagar Campus, Uttarakhand, India; ^2^ Department of Biotechnology, Goa University, Taleigao, India; ^3^ Department of Biological Sciences, KK Birla, Goa Campus, BITS Pilani, Zuarinagar, India

**Keywords:** Sertoli cell, germ cell, androgen receptor, retinoic acid, meiosis, spermatogenesis

## Abstract

Germ cells (Gc) propagate the genetic information to subsequent generations. Diploid (2n) Gc get transformed to specialized haploid (n) gametes by mitotic and meiotic divisions in adult gonads. Retinoic acid (RA), an active derivative of vitamin A (retinol), plays a critical role in organ morphogenesis and regulates the meiotic onset in developing Gc. Unlike ovaries, fetal testes express an RA-degrading enzyme CYP26B1, and thereby, male Gc fail to enter into meiosis and instead get arrested at G_0_/G_1_ stage, termed as gonocytes/pro-spermatogonia by embryonic (E) 13.5 days. These gonocytes are transformed into spermatogonial stem/progenitor cells after birth (1–3 days of neonatal age). During post-natal testicular maturation, the differentiating spermatogonia enter into the meiotic prophase under the influence RA, independent of gonadotropic (both FSH and LH) support. The first pulse of RA ensures the transition of undifferentiated type A spermatogonia to differentiated A_1_ spermatogonia and upregulates STRA8 expression in Gc. Whereas, the second pulse of RA induces the meiotic prophase by augmenting MEIOSIN expression in differentiated spermatogonia B. This opinion article briefly reviews our current understanding on the RA-driven spermatogonial differentiation in murine testes.

## Introduction

Spermatogenesis occurs within testes where male germ cells (Gc) undergo both mitotic and meiotic divisions to form haploid (n) spermatozoa (Griswold, 2016). This is a tightly regulated asynchronized process, broadly sub-divided into four major developmental stages, namely, 1) pre-meiotic proliferation of undifferentiated spermatogonia, 2) meiotic entry of differentiated spermatogonia 3) meiotic completion and formation of round spermatid, and 4) maturation of elongated spermatid to spermatozoa (Griswold, 2016). Testicular Sertoli cells (Sc) provide the structural and biochemical support to all the stages of male Gc within the seminiferous tubules (Griswold, 2018; Bhattacharya et al., 2019). Both Gc-specific intrinsic and Sc-derived extrinsic factors collectively determine the Gc developmental fate (Griswold, 2018; Bhattacharya et al., 2019).

Retinoic acid (RA) is the oxidized derivative/metabolite of vitamin A [retinol (ROL)] and plays a crucial role in pattern formation during organogenesis (Cunningham and Duester, 2015). In fetal ovaries (not in fetal testes), RA triggers the meiotic onset in developing Gc (Endo et al., 2019). During post-natal testicular maturation, RA induces the transition of undifferentiated spermatogonia A to differentiating spermatogonia A_1_ and governs the meiotic entry of differentiated spermatogonia B, and this action continues in successive spermatogenic waves (Griswold, 2016). Furthermore, RA also has been shown to be essential for maintaining the remodeling and permeability of blood–testis barrier, meiotic recombination and progression, and spermiation (Gewiss et al., 2020). We here briefly discuss our current understanding on the critical role of RA in regulating male Gc differentiation in fetal and post-natal testes considering mouse as a model organism.

## Biosynthesis of Retinoic Acid and Signaling

Vitamin A or ROL is synthesized in the liver and delivered to testes *via* a complex formed by retinol-binding protein 4 (RBP4) and transthyretin (TT4) (Gewiss et al., 2020). ROL is taken up by the membrane receptor STRA6 (stimulated by retinoic acid gene 6) expressed by Sc (Gewiss et al., 2020). The first-rate limiting step of RA production is the reversible conversion of ROL to retinal (RAL) by NAD^+^-dependent retinol dehydrogenase 10 (RDH10) expressed by Sc (Gewiss et al., 2020). The second step is an irreversible oxidation of RAL to RA catalyzed by the retinaldehyde dehydrogenase (s) (ALDH1A1, ALDH1A2, and ALDH1A3) expressed by both Sc and meiotic Gc (Gewiss et al., 2020).

**FIGURE 1:**
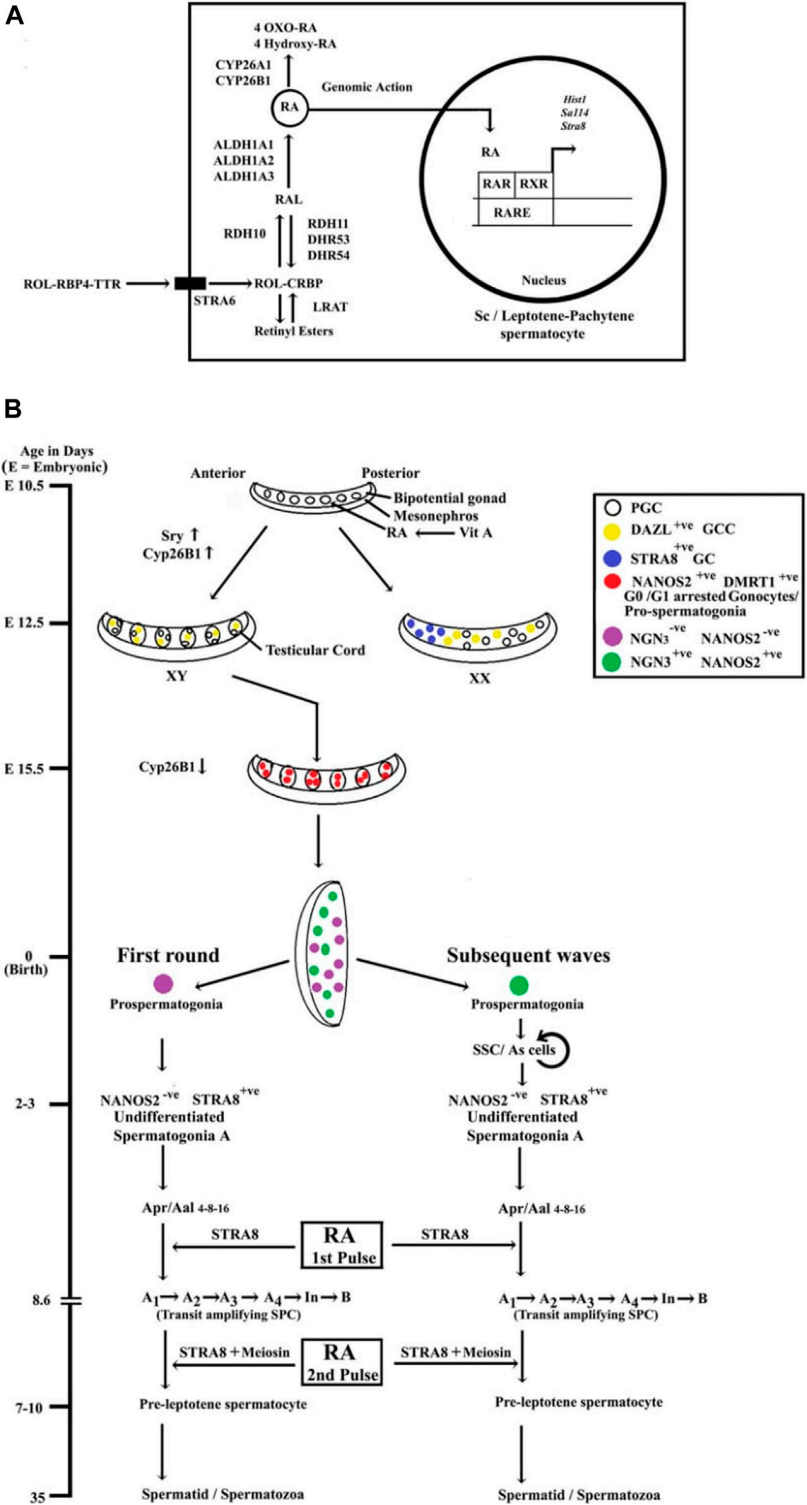
**(A)** The biosynthesis and signaling of RA in testicular Sc and/or meiotic Gc. Vitamin A or retinol (ROL) is synthesized in the liver and delivered to testes *via* retinol-binding protein 4 (RBP4) and transthyretin (TTR). ROL is uptaken by a membrane-bound receptor termed stimulated by retinoic acid gene 6 (STRA6) expressed by Sc. In Sc, lecithin retinol acyltransferase (LRAT) can convert ROL [bound with cellular retinol-binding protein (CRBP)] into retinyl esters, or ROL gets converted into retinal (RAL) by NAD^+^-dependent retinol dehydrogenase 10 (RDH10). The reverse step, i.e., conversion of RAL to ROL, is also mediated by NADPH-dependent retinal reductases (RDH11, DHRS3, and DHRS4). In both Sc and meiotic Gc, the second step, i.e., the irreversible oxidation of RAL to RA, is catalyzed by the retinaldehyde dehydrogenase(s) (ALDH1A1, ALDH1A2, and ALDH1A3). RA gets degraded and further oxidized to 4-hydroxy RA/4-oxo-RA by CYP26A1 or CYP26B1 enzymes. RA signaling involves two families of nuclear hormone receptors, namely, the retinoic acid receptors (RARs) and the retinoid X receptors (RXRs). In target cells, RA-activated RAR-RXR heterodimers bind to retinoic acid response elements (RAREs) located in the cis-acting regulatory sequences of the RA-responsive genes (*Stra8*, *Hist1*, *Sall4*, etc.) and induce/modulate their transcriptions. **(B)** Summary of the developmental time schedule of male Gc development and the cellular target sites of RA actions in murine testes. Retinoic acid (RA) is produced by embryonic mesonephros and diffused to the adjacent bipotential gonad establishing an anterior to posterior gradient. During embryonic (E) 10.5–12.5 days, primordial germ cells (PGC) get transformed into gametogenesis-competent cells (GCC) by induction of RNA-binding proteins *Dazl*. In XY gonad, which has been differentiated into fetal testis by *Sry* during E 11.5–12.5 days, RA gets degraded by an enzyme CYP26B1. By E 13.5 days, the Gc get arrested into G_0_/G_1_ stage and termed as gonocytes/pro-spermatogonia until birth (day 0). Although the expression of CYP26B1 gets downregulated in fetal testes by E 13.5 days, Gc are maintained at this quiescent stage (G_0_/G_1_ stage) of pro-spermatogonia *via* destabilizing DAZL by an intrinsic factor NANOS2. By post-natal days 2–3, the SSC (designated A_s_ spermatogonia) system gets established from neurogenin 3 (NGN3)^+^ lineage with specific markers, e.g., ID4, GFRa1, NANOS2, etc. Whereas, the first round of differentiated spermatogonia appears from a unique NGN3^−^, NANOS2^−/low^ lineage of pro-spermatogonia, independent from the SSC pool and directly form the STRA8^+^ A_1_ spermatogonial cells. During subsequent spermatogenic waves, STRA8^+^ A_1_ spermatogonia originate from the NGN3^+^, NANOS2^+^ SSC. The first pulse of RA from Sc (by 2 days of post-natal age) induces the formations of the transit-amplifying spermatogonial progenitor cells (SPC) including A_1_, A_2_, A_3_, A_4_, intermediate (In), and B spermatogonia population and upregulates STRA8 expression, covering 8.6 days. The second pulse of RA in pre-pubertal testes augments both STRA8 and MEIOSIN, which act as the gatekeepers of meiotic entry for differentiated spermatogonia B.

RA signaling involves two families of nuclear hormone receptors, namely, the retinoic acid receptors (RARs) and the retinoid X receptors (RXRs), each having three isotypes [RARα, RARβ, and RARγ or RXRα, RXRβ, and RXRγ (Endo et al., 2019; Gewiss et al., 2020)]. In target cells, ligand (i.e., RA)-activated RAR-RXR heterodimers bind to retinoic acid response elements (RAREs) located in the cis-acting regulatory sequences of the RA-responsive genes and induce/modulate their transcriptions (Endo et al., 2019; Gewiss et al., 2020). Figure 1A shows the biosynthesis and signaling of cascade RA.

## Origin and Migration of Germ Line Cells in Fetal Testes

In mice, primordial germ cells (PGC) originate from epiblast at embryonic age (E) 6.5 days, whereas the somatic genital ridge (GR) is formed from the coelomic epithelium of the mesonephros by E 8.5–9.5 days (Endo et al., 2019). PGC migrate from the base of the allantois to the GR, and this process is completed by E 10.5–11 days (Endo et al., 2019). Migratory PGC are mitotically active expressing pluripotency markers (e.g., OCT4, SOX2, and NANOG) and can form teratomas (Endo et al., 2019). However, after the arrival at the GR/bipotential gonad, PGC undergo a massive global demethylation during E 10.5–12.5 days (Seisenberger et al., 2012). This extensive epigenetic reprogramming leads to the induction of RNA-binding proteins *Dazl* (deleted in azoospermia-like) and *Ddx4* (DEAD box polypeptide 4, also called mouse vasa homolog), and PGC get transformed into gametogenesis-competent cells (GCC) (Lin et al., 2008). GATA-4 plays a critical role in the formation of somatic GR and substantially supports the licensing of PGC to be responsive towards RA signal (Hu et al., 2015). On E 11.5 days, *Sry* gene gets activated in SF-1/Nr5a1^+^ somatic cells of XY embryos, induces the genetic cascade of male programming genes (*Sox9*, *Amh*, *Fgf9*, *Dmrt1*, etc.) essential for sex determination, and initiates the testicular morphogenesis by influx of mesonephric endothelial/perivascular cells to XY gonads to form male-specific vasculature pattern and eventually testicular cord (Koopman et al., 1990; Martineau et al., 1997; Svingen and Koopman, 2013; DeFalco et al., 2014; Stévant et al., 2018). By E 12.5 days, the fetal Sc are completely specified with SOX9 as molecular marker in fetal testis (Svingen and Koopman, 2013).

## Degradation of Retinoic Acid in Fetal Testes

RA is produced by adjacent mesonephros and diffuses through the fetal gonad, establishing a robust anterior-to-posterior gradient (Endo et al., 2019). Under the influence of RA, the GCC enter into meiotic prophase by upregulating transcription factor *Stra8* (stimulated by retinoic acid gene 8) and *Rec 8* (coding for REC8 meiotic recombination protein) in the XX gonads, which have been specified as fetal ovary by E 12.5 days (Bowles et al., 2006; Koubova et al., 2006; Endo et al., 2019). However, in XY gonad, which is differentiated into fetal testis, male GCS fail to initiate the meiotic prophase due to the degradation of RA by CYP26B1 (Griswold, 2016; Endo et al., 2019). Ectopic expression of STRA8^+^ meiotic Gc has been observed in CYP26B1-deficient male embryos, confirming the role of RA in inducing Gc meiosis in males (MacLean et al., 2007). By E 13.5 days of age, male Gc get arrested into G_0_/G_1_ stage and termed as gonocytes/pro-spermatogonia (Griswold, 2016; Du et al., 2021). However, although the expression of CYP26B1 gets downregulated in fetal testes by E 13.5 days, Gc are maintained at this quiescent stage (G_0_/G_1_ stage) of pro-spermatogonia *via* destabilizing DAZL by Gc intrinsic factor NANOS2 (Griswold, 2016; Endo et al., 2019; Du et al., 2021). It is important here to note that in *Nanos2*-null XY embryos, Gc initiate ectopic meiosis by expressing STRA8 by E14.5 (Suzuki and Saga, 2008).

## Spermatogonial Stem Cell or Spermatogonial Progenitor Cell Population in Neonatal Testes

The pro-spermatogonia or gonocytes form the spermatogonial stem cell (SSC) or spermatogonial progenitor cell (SPC) population by 1–2 days post-birth (Lord and Oatley, 2017; Yoshida, 2018). By post-natal age of 2–3 days, the SSC (designated A_s_ spermatogonia) and/or SPC (undifferentiated spermatogonia A) system get established with specific markers for self-renewal (e.g., ID4, PAX7, RHOX5, BMI1, EOMES, GFRA1, NANOS2, UTF1, ZBTB16, SALL4, LIN28, FOXO1, *miR-21*, and *mirR-17∼92*/*miRc1*) (Lord and Oatley, 2017; Kubota and Brinster, 2018; Yoshida, 2018). The specific topological location of the SSC pool and its unique micro-environment restrict the direct action of RA on SSC (Lord and Oatley, 2017; Kubota and Brinster, 2018; Yoshida, 2018). During the initiation of the spermatogonial differentiation, the SSC/SPC first downregulates these self-renewal-associated genes [*Id4*, *Gfra1*, *Nanos2*, *Pou5f1*, *Zbtb16* (*Plzf*), *Lin28*, etc.] and upregulates the genes necessary for differentiation like *cKit-receptor*, *Sohlh1/2*, *Stra8*, *Ccnd2*, and *Sall4* (Griswold, 2016; Lord and Oatley, 2017; Kubota and Brinster, 2018; Yoshida, 2018). Notably, the first round of differentiated spermatogonia appears from a unique neurogenin 3 (NGN3)^−^, NANOS2^−/low^ lineage of pro-spermatogonia, independent from the SSC/SPC pool and directly form the STRA8^+^ A_1_ spermatogonial cells (Griswold, 2016; Yoshida, 2018). However, during subsequent spermatogenic waves, STRA8^+^ A_1_ spermatogonia originate from the NGN3^+^ SSC/SPC cells (Griswold, 2016; Yoshida, 2018). Data from *hpg* mouse (lacking endogenous GnRH) or mutant mice models having altered/ablated gonadotropic functions demonstrate that the initial expansion/differentiation of the spermatogonial population is not dependent on either FSH or LH (Kumar, 2007; Jonas et al., 2014). However, a significant shift in FSH action with dominant cAMP signaling has been shown to be associated with the rapid transition of spermatogonia A to B in maturing rat Sc between 9 and 12 days of post-natal age (Bhattacharya et al., 2012).

## The Pulses of Retinoic Acid in Neonatal/Pre-Pubertal Testes

Historically, in vitamin A-deficient (VAD) rodents, the transition of the undifferentiated spermatogonia A into differentiating spermatogonia A_1_ has been found to be blocked, and exogenous supplementations of RA in VAD animals restore the initiation of spermatogonial differentiation (Wolbach and Howe, 1925; Mitranond et al., 1979; Morales and Griswold, 1987; van Pelt and de Rooij, 1990; McLean et al., 2002). Pharmacological inhibition of the conversion of RAL to RA by bis-(dichloroacetyl)-diamines (BDAD) compounds like WIN 18446 and/or genetic ablations of the RA synthesizing enzymes have confirmed that RA triggers the differentiation of spermatogonial cells in rodents (Griswold, 2016; Gewiss et al., 2020). RA acts on the differentiating spermatogonia A *via* both classical genomic or non-genomic pathways to induce 1) genes required for G1/S phase transition (e.g., *Hist1* and *Ccnd2*), 2) genes critical for initiating spermatogonial differentiation (*c-Kit* and *Sall4*), and 3) genes to promote the meiotic prophase [*Stra8* (transcription factor inducing meiotic entry); *Rec8* (a component of the cohesin complex critical for chromosomal segregation and synapsis); and subsequently *Dmc1, Sycp3*, etc.] (Chen et al., 2016). Very recently, another transcription factor having helix-loop-helix (HLH) and high-mobility-group (HMG) domain termed as MEIOSIN has been shown to be a crucial regulator of meiotic initiation (Ishiguro et al., 2020; Oatley and Griswold, 2020). Although both STRA8 and MEIOSIN are induced by RA signaling, the timing of such inductions differs (Oatley and Griswold, 2020). The transcription of *Stra8* appears during the transition of A_al_ to A_1_ stage with first pulse of RA from Sc (by 2 days of post-natal age), but the Gc do not directly switch over to meiotic prophase and rather continue with six additional mitotic divisions creating the transit-amplifying SPC [A_1_, A_2_, A_3_, A_4_, intermediate (In), and B spermatogonia] population (Griswold, 2016; Gewiss et al., 2020). These six mitotic divisions occur over a span of exactly 8.6 days in mice testes (Griswold, 2016). During spermatogonial differentiation, RA signaling directly regulates the expression of replication-dependent core histone genes that is critical for entering into S phase (Chen et al., 2016). However, the second pulse of RA induces the transcription of *Meiosin* and induces the mitosis-to-meiosis switch in B spermatogonia to form pre-leptotene spermatocytes in pre-pubertal testes (Oatley and Griswold, 2020). It is important to note here that, despite an accumulation of undifferentiated spermatogonia and pre-leptotene spermatocyte arrest, the transition of A_al_ to A_1_ stage remains unaffected in *Stra8* or *Meiosin* null mice (Endo et al., 2015; Ishiguro et al., 2020). Therefore, RA can be termed as the “driver” whereas *Stra8* or *Meiosin* as gatekeeper/watchman for meiotic onset.

## Source of Retinoic Acid

RA employs both juxtracrine/paracrine and autocrine mode of action in testes (Griswold, 2016; Gewiss et al., 2020). Both Sc-derived extrinsic and Gc-specific intrinsic RA collectively determine the Gc developmental fate in adult testes (Griswold, 2016; Gewiss et al., 2020). Although the synthesis of RA occurs in both Sc and pachytene/diplotene spermatocytes (during stages VII–XIII), Sc are the primary site of RA signaling and direct the upregulation of STRA8 during the first round of spermatogenesis (Griswold, 2016; Gewiss et al., 2020). Sc-specific ablation of either *Rdh10* or *Aldh1a1-3* leads to a complete block of the transition of spermatogonia A to A_1_ stage (Griswold, 2016; Gewiss et al., 2020). Intriguingly, in Sc-specific *Rdh10* mutant mice, Gc do compensate the need of RA as spermatogenesis gets naturally restored in the next round (after 30 days) without any exogenous support of RA (Tong et al., 2013). However, mice with Sc-specific ablation of *Aldh1a1*, *Aldh1a2*, and *Aldh1a3* require the external stimulation of RA to recover and maintain the spermatogenic continuation (Raverdeau et al., 2012). This data confirmed that RA synthesized/supplied by Sc is sufficient to drive only the first round of spermatogenesis, whereas the Gc intrinsic RA is critically essential for spermatogenic maintenance in subsequent waves (Griswold, 2016; Gewiss et al., 2020). Notably, the lack or unavailability of RA results in the accumulation of SSC or SPC, whereas a high concentration of RA shows no adverse impact on male fertility (Griswold, 2016; Gewiss et al., 2020). Interestingly in post-natal testis, RA-degrading enzymes CYP26A1, CYP26B1, and CYP26C1 are expressed in peritubular myoid cells (PTc) and Gc but not in steroidogenic Leydig cells (Lc) (Gewiss et al., 2020). Gc- or Sc-specific single/double knock-out (KO) mice for *Cyp26a1* and *Cyp26b1* result into male sub-fertility, confirming the critical requirement of the patchy, non-uniform pulse of RA within the seminiferous tubules in an asynchronous manner for optimal spermatogenesis (Hogarth et al., 2015; Griswold, 2016; Gewiss et al., 2020). It is also important to note that a critical suppressor of RA action on Gc is DMRT1 (double-sex and mab-3-related transcription factor 1) (Matson et al., 2010). Gc-specific *Dmrt1* mutant shows precocious induction of STRA8 followed by meiotic entry (Matson et al., 2010).

## Concluding Remarks

The pharmacological inhibition of RA biosynthesis and/or *in vivo* genetic ablations of RA-synthesizing enzymes have confirmed the critical role of RA signaling in regulating male Gc fate decisions. Fetal Sc express CYP26B1 and thereby restrict the action of RA to induce the meiotic onset in male GCG and ensure the formation of mitotically quiescent pro-spermatogonia. Both NANOS2 and DMRT1 act as essential Gc intrinsic factors to prevent the meiotic entry of pro-spermatogonia in late gestation period (after E 13.5 days) and early neonatal age (first 7 days after birth). In post-natal testes, the pulses of RA drives 1) the conversion of undifferentiated spermatogonia A to differentiated A_1_ stage, 2) the expansion of the transit-amplifying SPC population (spermatogonia A_1_ to spermatogonia B), and finally 3) the induction of meiotic prophase to form the pre-leptotene spermatocytes. Figure 1B summarizes the developmental time schedule of male Gc and the specific cellular targets of RA in murine testes.

## Future Directions

Despite sincere efforts from various groups, limited RA-responsive gene(s) (e.g., *Stra8*, *Rec8*, *c-Kit*, *Hist1*, *Sall4*, and *Meiosin*) having potential/key role(s) in regulating the meiotic onset have been identified up to date in murine testes (Oatley and Griswold, 2020). Therefore, RA-mediated regulatory genetic networks critical for the induction of spermatogonial differentiation and determining the developmental switch for the transition of Gc from mitosis to meiosis are largely unknown (Tan and Wilkinson, 2020). Future studies are thereby required to investigate the in depth-molecular landscape associated with RA signaling cascade in purified spermatogonial cells employing advanced single-cell transcriptomic analyses (Suzuki et al., 2019).
